# 2-Amino-5-nitro­pyridinium hydrogen oxalate

**DOI:** 10.1107/S160053681400525X

**Published:** 2014-03-26

**Authors:** M. Ambrose Rajkumar, S. Stanly John Xavier, S. Anbarasu, Prem Anand Devarajan, M. NizamMohideen

**Affiliations:** a Physics Research Centre, Department of Physics, St Xavier’s College (Autonomous), Palayamkottai 627 002, Tamil Nadu, India; b Department of Chemistry, St Xavier’s College (Autonomous), Palayamkottai 627 002, Tamil Nadu, India; c Department of Physics, The New College (Autonomous), Chennai 600 014, Tamil Nadu, India

## Abstract

In the cation of the title mol­ecular salt, C_5_H_6_N_3_O_2_^+^·C_2_HO_4_^−^, the dihedral angle between the aromatic ring and the nitro group is 3.5 (3)°; in the anion, the dihedral angle between the CO2 and CO_2_H planes is 10.5 (2)°. In the crystal, the anions are linked into [100] chains by O—H⋯O hydrogen bonds. The cations cross-link the chains by way of N—H⋯O hydrogen bonds and the structure is consolidated by C—H⋯O inter­actions.

## Related literature   

For the crystal structures of related pyridine derivatives, see: Babu *et al.* (2014 ▶); Anderson *et al.* (2005 ▶); Karle *et al.* (2003 ▶). For simple organic–inorganic salts containing strong inter­molecular hydrogen bonds, see: Fu *et al.* (2011 ▶); Sethuram *et al.* (2013*a* ▶,*b* ▶); Shihabuddeen Syed *et al.* (2013 ▶); Showrilu *et al.* (2013 ▶); Huq *et al.* (2013 ▶). For the structure of oxalic acid, see: Derissen & Smith (1974 ▶). For graph-set analysis, see: Bernstein *et al.*(1995 ▶). 
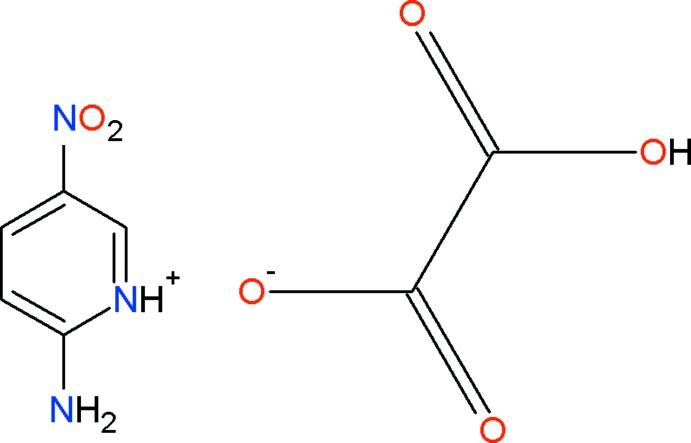


## Experimental   

### 

#### Crystal data  

C_5_H_6_N_3_O_2_^+^·C_2_HO_4_^−^
*M* *_r_* = 229.16
Triclinic, 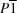

*a* = 5.5609 (2) Å
*b* = 9.2012 (4) Å
*c* = 9.2305 (4) Å
α = 90.245 (2)°
β = 98.500 (2)°
γ = 100.038 (2)°
*V* = 459.74 (3) Å^3^
*Z* = 2
Mo *K*α radiation
μ = 0.15 mm^−1^
*T* = 293 K
0.35 × 0.30 × 0.30 mm

#### Data collection  

Bruker Kappa APEXII CCD diffractometer
Absorption correction: multi-scan (*SADABS*; Sheldrick, 2004 ▶) *T*_min_ = 0.950, *T*_max_ = 0.957
10142 measured reflections
1615 independent reflections
1417 reflections with *I* > 2σ(*I*)
*R*_int_ = 0.020

#### Refinement  

*R*[*F* ^2^ > 2σ(*F* ^2^)] = 0.039
*wR*(*F* ^2^) = 0.110
*S* = 1.07
1615 reflections
153 parameters
H atoms treated by a mixture of independent and constrained refinement
Δρ_max_ = 0.28 e Å^−3^
Δρ_min_ = −0.32 e Å^−3^

### 

Data collection: *APEX2* (Bruker, 2004 ▶); cell refinement: *APEX2* and *SAINT* (Bruker, 2004 ▶); data reduction: *SAINT* and *XPREP* (Bruker, 2004 ▶); program(s) used to solve structure: *SHELXS97* (Sheldrick, 2008 ▶); program(s) used to refine structure: *SHELXL97* (Sheldrick, 2008 ▶); molecular graphics: *ORTEP-3 for Windows* (Farrugia, 2012 ▶) and *Mercury* (Macrae *et al.*, 2008 ▶); software used to prepare material for publication: *WinGX* (Farrugia, 2012 ▶) and *PLATON* (Spek, 2009 ▶).

## Supplementary Material

Crystal structure: contains datablock(s) global, I. DOI: 10.1107/S160053681400525X/jj2183sup1.cif

Structure factors: contains datablock(s) I. DOI: 10.1107/S160053681400525X/jj2183Isup2.hkl

Supporting information file. DOI: 10.1107/S160053681400525X/jj2183Isup3.cml

CCDC reference: 990527

Additional supporting information:  crystallographic information; 3D view; checkCIF report

## Figures and Tables

**Table 1:** Hydrogen-bond geometry (Å, °)

*D*—H⋯*A*	*D*—H	H⋯*A*	*D*⋯*A*	*D*—H⋯*A*
C2—H2⋯O5^i^	0.93	2.48	3.323 (2)	152
C3—H3⋯O2^ii^	0.93	2.37	3.296 (2)	178
C5—H5⋯O1^iii^	0.93	2.42	3.186 (2)	140
C5—H5⋯O4^iv^	0.93	2.44	2.970 (2)	116
N1—H1⋯O6^iv^	0.86	1.94	2.7697 (18)	160
O4—H4⋯O5^v^	0.82	1.64	2.4486 (16)	170
N2—H2*A*⋯O3^v^	0.89 (3)	2.16 (3)	2.959 (2)	149 (2)
N2—H2*A*⋯O5^v^	0.89 (3)	2.36 (3)	3.007 (2)	130 (2)
N2—H2*B*⋯O3^vi^	0.89 (3)	1.99 (3)	2.870 (2)	173 (2)

Symmetry codes: (i) 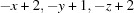; (ii) 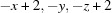; (iii) 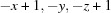; (iv) 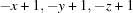; (v) 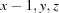; (vi) 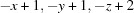.

**Fig. 1.**
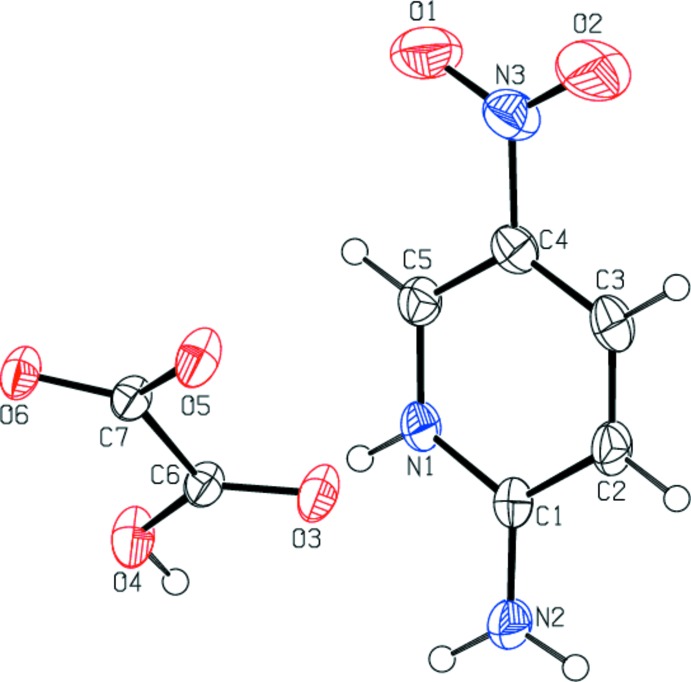
View of the title compound showing the atom-numbering scheme. Displacement ellipsoids are drawn at the 40% probability level. H atoms are presented as a small spheres of arbitrary radius.

**Fig. 2.**
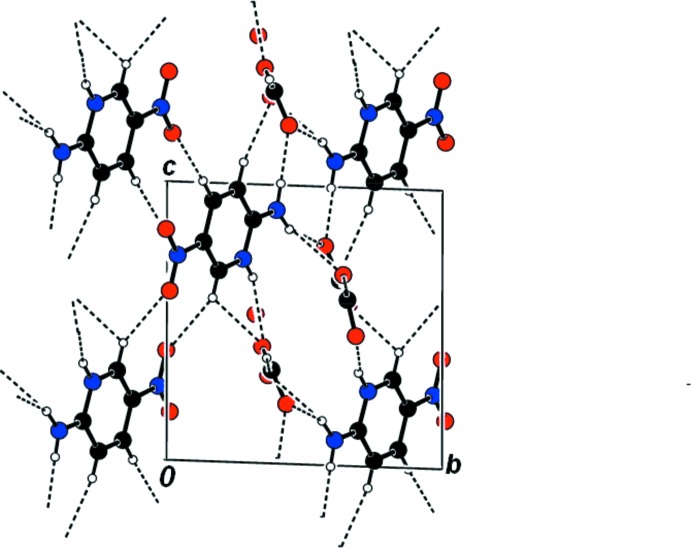
The crystal packing of the title compound viewed along a axis. N—H···O, O—H···O hydrogen bonds and weak C—H···O intermolecular inteeractions are shown as dashed lines, forming a three-dimensional network. H atoms not involved in hydrogen bonding have been omitted for clarity.

## supplementary crystallographic information

### 1. Comment

Simple organic–inorganic salts containing strong intermolecular hydrogen bonds have attracted an attention as materials which display ferroelectric-paraelectric phase transitions (Fu *et al.*, 2011; Sethuram, *et al.*, 2013*a*,*b*; Huq, *et al.*, 2013; Shihabuddeen Syed, *et al.*, 2013; Showrilu, *et al.*, 2013). As part of our ongoing investigations of pyridine derivatives (Babu *et al.*, 2014), the title compound was synthesized and we report herein on its crystal structure.

In the title salt, (C_5_H_6_N_3_O_2_)^+^, (C_2_HO_4_)^-^, the asymmetric unit consists of an independent 2-amino-5-nitropyridinium cation, and oxalic actetate anion, which lie on an inversion symmetry. A proton transfer from the carboxyl group of oxalic acid to atom N1 of 2-amino-5-nitro pyridinine resulted in the formation of a salt. This protonation lead to the widening of the C5-N1-C1 angle of the pyridine ring to 122.79 (14)°, compared to 115.25 (13)° in the unprotonated aminopyridine (Anderson *et al.*, 2005). This type of protonation is observed in various aminopyridine acid complexes (Babu *et al.*, 2014; Karle *et al.*, 2003).

The bond lengths and bond angles of the aminopyridine are comparable to the values reported earlier for aminopyridine (Babu *et al.*, 2014; Anderson *et al.*, 2005). The bond lengths and bond angles of the oxalate are comparable to the values reported for oxalic acid (Derissen & Smith, 1974). The non hydrogen pyridine ring, C1/C2/C3/C4/C5/N1, is planar with a maximum deviation of 0.006 (1)Å from the least squares plane for the C3 atom, with the endocyclic angles covering range of 117.88 (16) - 122.79 (14)°. The hydrogen oxalate anion O3/O4/O5/O6/C6/C7, is less planar with a maximum deviation of -0.131 (1)Å for the O3 and O6 atoms.

The crystal packing is consolidated by intermolecular N—H···O and O—H···O hydrogen bonds and weak C—H···O intermolecular interactions (Table 1 and Fig. 2). In the crystal structure, the 2-Amino-5-nitropyridinium unit is bound to acetate anions by five distinct N—H···O hydrogen bonds. The ion pairs are joined by two N—H···O hydrogen bonds in which the N atom of the 2-amino-5-nitroPyridinium unit acts as a bifurcated donor, thus generating *R*_1_^2^(5) ring motifs (Bernstein *et al.*, 1995). The hydroxyl group hydrogen atom is also hydrogen-bonded to the carboxylate oxygen atom through strong intermolecular O—H···O hydrogen bonds, with the O···O distance of 2.4486 (16)Å, which is from a chain, C(5), running along the *b* axis (Bernstein *et al.*, 1995). The structure is further stabilized by weak C—H···O intermolecular inteactions, forming a three-dimensional network.

### 2. Experimental

Crystals of the title compound were obtained by slow evaporation of a 1:1 mol. mixture of 2-amino-5-nitropyridine and oxalic acid in methanol at room temperature.

### 3. Refinement

The amino group NH_2_ H atoms of the pyridine derivatives were located in difference Fourier maps and refined in the riding mode approximation. The OH, NH(Protonated) and C-bound H-atoms were placed in calculated positions and treated as riding atoms: O-H = 0.82Å, N-H = 0.86Å, C-H = 0.93Å with Uiso(H) = 1.5Ueq(O) and = 1.2Ueq(N,C) for other H atoms.

### Crystal data

**Table:**

C_5_H_6_N_3_O_2_^+^·C_2_HO_4_^−^	*Z* = 2
*M**_r_* = 229.16	*F*(000) = 236
Triclinic, *P*1	*D*_x_ = 1.655 Mg m^−^^3^
Hall symbol: -P 1	Mo *K*α radiation, λ = 0.71073 Å
*a* = 5.5609 (2) Å	Cell parameters from 2794 reflections
*b* = 9.2012 (4) Å	θ = 2.4–31.1°
*c* = 9.2305 (4) Å	µ = 0.15 mm^−^^1^
α = 90.245 (2)°	*T* = 293 K
β = 98.500 (2)°	Block, colourless
γ = 100.038 (2)°	0.35 × 0.30 × 0.30 mm
*V* = 459.74 (3) Å^3^	

### Data collection

**Table:**

Bruker Kappa APEXII CCD diffractometer	1615 independent reflections
Radiation source: fine-focus sealed tube	1417 reflections with *I* > 2σ(*I*)
Graphite monochromator	*R*_int_ = 0.020
Detector resolution: 0.1000 pixels mm^-1^	θ_max_ = 25.0°, θ_min_ = 2.2°
ω and φ scans	*h* = −6→6
Absorption correction: multi-scan (*SADABS*; Sheldrick, 2004)	*k* = −10→10
*T*_min_ = 0.950, *T*_max_ = 0.957	*l* = −10→10
10142 measured reflections	

### Refinement

**Table:**

Refinement on *F*^2^	Primary atom site location: structure-invariant direct methods
Least-squares matrix: full	Secondary atom site location: difference Fourier map
*R*[*F*^2^ > 2σ(*F*^2^)] = 0.039	Hydrogen site location: inferred from neighbouring sites
*wR*(*F*^2^) = 0.110	H atoms treated by a mixture of independent and constrained refinement
*S* = 1.07	*w* = 1/[σ^2^(*F*_o_^2^) + (0.0559*P*)^2^ + 0.2329*P*] where *P* = (*F*_o_^2^ + 2*F*_c_^2^)/3
1615 reflections	(Δ/σ)_max_ < 0.001
153 parameters	Δρ_max_ = 0.28 e Å^−^^3^
0 restraints	Δρ_min_ = −0.32 e Å^−^^3^

### Special details

**Table:**

Geometry. All esds (except the esd in the dihedral angle between two l.s. planes) are estimated using the full covariance matrix. The cell esds are taken into account individually in the estimation of esds in distances, angles and torsion angles; correlations between esds in cell parameters are only used when they are defined by crystal symmetry. An approximate (isotropic) treatment of cell esds is used for estimating esds involving l.s. planes.
Refinement. Refinement of F^2^ against ALL reflections. The weighted R-factor wR and goodness of fit S are based on F^2^, conventional R-factors R are based on F, with F set to zero for negative F^2^. The threshold expression of F^2^ > 2sigma(F^2^) is used only for calculating R-factors(gt) etc. and is not relevant to the choice of reflections for refinement. R-factors based on F^2^ are statistically about twice as large as those based on F, and R- factors based on ALL data will be even larger.

### Fractional atomic coordinates and isotropic or equivalent isotropic displacement parameters (Å^2^)

**Table:**

	*x*	*y*	*z*	*U*_iso_*/*U*_eq_	
C1	0.3486 (3)	0.30577 (19)	0.87248 (18)	0.0302 (4)	
C2	0.5136 (3)	0.2468 (2)	0.97783 (19)	0.0361 (4)	
H2	0.5206	0.2695	1.0768	0.043*	
C3	0.6606 (3)	0.1580 (2)	0.9356 (2)	0.0380 (4)	
H3	0.7711	0.1201	1.0046	0.046*	
C4	0.6446 (3)	0.12349 (19)	0.78639 (19)	0.0338 (4)	
C5	0.4879 (3)	0.18053 (19)	0.68547 (19)	0.0332 (4)	
H5	0.4796	0.1582	0.5863	0.040*	
C6	0.8206 (3)	0.61691 (18)	0.66445 (17)	0.0270 (4)	
C7	1.0607 (3)	0.64305 (19)	0.59536 (17)	0.0269 (4)	
N1	0.3441 (3)	0.26987 (16)	0.72996 (15)	0.0323 (4)	
H1	0.2451	0.3057	0.6649	0.039*	
N2	0.2009 (3)	0.39211 (19)	0.90933 (19)	0.0408 (4)	
N3	0.7996 (3)	0.02858 (19)	0.73614 (19)	0.0450 (4)	
O1	0.7803 (3)	−0.0001 (2)	0.60645 (19)	0.0674 (5)	
O2	0.9490 (4)	−0.0148 (3)	0.8270 (2)	0.0882 (7)	
O3	0.8197 (2)	0.56345 (16)	0.78510 (13)	0.0410 (4)	
O4	0.6388 (2)	0.65291 (16)	0.58166 (14)	0.0409 (4)	
H4	0.5159	0.6372	0.6226	0.061*	
O5	1.2521 (2)	0.62615 (16)	0.68401 (13)	0.0395 (4)	
O6	1.0537 (2)	0.67353 (14)	0.46624 (12)	0.0342 (3)	
H2A	0.114 (5)	0.439 (3)	0.842 (3)	0.060 (7)*	
H2B	0.208 (4)	0.407 (2)	1.005 (3)	0.051 (6)*	

### Atomic displacement parameters (Å^2^)

**Table:**

	*U*^11^	*U*^22^	*U*^33^	*U*^12^	*U*^13^	*U*^23^
C1	0.0316 (9)	0.0336 (9)	0.0242 (8)	0.0042 (7)	0.0024 (7)	0.0058 (7)
C2	0.0424 (10)	0.0428 (10)	0.0217 (8)	0.0085 (8)	−0.0013 (7)	0.0051 (7)
C3	0.0379 (10)	0.0426 (10)	0.0313 (9)	0.0111 (8)	−0.0070 (7)	0.0093 (8)
C4	0.0324 (9)	0.0333 (9)	0.0351 (10)	0.0082 (7)	0.0002 (7)	0.0039 (7)
C5	0.0387 (10)	0.0347 (9)	0.0253 (9)	0.0074 (7)	0.0010 (7)	0.0015 (7)
C6	0.0212 (8)	0.0392 (9)	0.0216 (8)	0.0085 (7)	0.0027 (6)	0.0034 (6)
C7	0.0205 (8)	0.0390 (9)	0.0221 (8)	0.0082 (6)	0.0022 (6)	0.0029 (6)
N1	0.0344 (8)	0.0391 (8)	0.0231 (7)	0.0118 (6)	−0.0027 (6)	0.0064 (6)
N2	0.0464 (10)	0.0537 (10)	0.0275 (9)	0.0224 (8)	0.0064 (7)	0.0078 (7)
N3	0.0458 (10)	0.0423 (9)	0.0481 (11)	0.0169 (8)	−0.0003 (8)	0.0007 (7)
O1	0.0832 (12)	0.0683 (11)	0.0567 (10)	0.0355 (9)	0.0044 (9)	−0.0148 (8)
O2	0.0942 (14)	0.1202 (17)	0.0668 (12)	0.0807 (14)	−0.0069 (10)	0.0069 (11)
O3	0.0313 (7)	0.0720 (9)	0.0249 (7)	0.0190 (6)	0.0090 (5)	0.0143 (6)
O4	0.0193 (6)	0.0712 (9)	0.0359 (7)	0.0150 (6)	0.0074 (5)	0.0221 (6)
O5	0.0192 (6)	0.0751 (10)	0.0257 (6)	0.0139 (6)	0.0014 (5)	0.0102 (6)
O6	0.0257 (6)	0.0560 (8)	0.0234 (6)	0.0122 (5)	0.0054 (5)	0.0099 (5)

### Geometric parameters (Å, º)

**Table:**

C1—N2	1.315 (2)	C6—O3	1.220 (2)
C1—N1	1.351 (2)	C6—O4	1.268 (2)
C1—C2	1.413 (2)	C6—C7	1.545 (2)
C2—C3	1.347 (3)	C7—O6	1.222 (2)
C2—H2	0.9300	C7—O5	1.2759 (19)
C3—C4	1.398 (3)	N1—H1	0.8600
C3—H3	0.9300	N2—H2A	0.89 (3)
C4—C5	1.352 (2)	N2—H2B	0.89 (3)
C4—N3	1.448 (2)	N3—O1	1.210 (2)
C5—N1	1.344 (2)	N3—O2	1.211 (2)
C5—H5	0.9300	O4—H4	0.8200
			
N2—C1—N1	119.96 (16)	O3—C6—C7	120.04 (14)
N2—C1—C2	122.16 (16)	O4—C6—C7	112.84 (13)
N1—C1—C2	117.88 (16)	O6—C7—O5	126.27 (14)
C3—C2—C1	120.31 (16)	O6—C7—C6	120.06 (14)
C3—C2—H2	119.8	O5—C7—C6	113.64 (13)
C1—C2—H2	119.8	C5—N1—C1	122.79 (14)
C2—C3—C4	118.97 (16)	C5—N1—H1	118.6
C2—C3—H3	120.5	C1—N1—H1	118.6
C4—C3—H3	120.5	C1—N2—H2A	121.5 (16)
C5—C4—C3	120.75 (17)	C1—N2—H2B	115.1 (15)
C5—C4—N3	118.45 (16)	H2A—N2—H2B	123 (2)
C3—C4—N3	120.79 (16)	O1—N3—O2	123.01 (19)
N1—C5—C4	119.29 (16)	O1—N3—C4	119.28 (16)
N1—C5—H5	120.4	O2—N3—C4	117.68 (17)
C4—C5—H5	120.4	C6—O4—H4	109.5
O3—C6—O4	127.10 (15)		
			
N2—C1—C2—C3	179.55 (17)	O3—C6—C7—O5	−10.4 (2)
N1—C1—C2—C3	0.1 (3)	O4—C6—C7—O5	171.21 (16)
C1—C2—C3—C4	−0.9 (3)	C4—C5—N1—C1	−0.1 (3)
C2—C3—C4—C5	1.3 (3)	N2—C1—N1—C5	−179.04 (16)
C2—C3—C4—N3	179.89 (17)	C2—C1—N1—C5	0.4 (3)
C3—C4—C5—N1	−0.8 (3)	C5—C4—N3—O1	−1.9 (3)
N3—C4—C5—N1	−179.43 (16)	C3—C4—N3—O1	179.50 (18)
O3—C6—C7—O6	168.06 (17)	C5—C4—N3—O2	175.9 (2)
O4—C6—C7—O6	−10.3 (2)	C3—C4—N3—O2	−2.7 (3)

### Hydrogen-bond geometry (Å, º)

**Table:**

*D*—H···*A*	*D*—H	H···*A*	*D*···*A*	*D*—H···*A*
C2—H2···O5^i^	0.93	2.48	3.323 (2)	152
C3—H3···O2^ii^	0.93	2.37	3.296 (2)	178
C5—H5···O1^iii^	0.93	2.42	3.186 (2)	140
C5—H5···O4^iv^	0.93	2.44	2.970 (2)	116
N1—H1···O4^iv^	0.86	2.47	2.9770 (18)	119
N1—H1···O6^iv^	0.86	1.94	2.7697 (18)	160
O4—H4···O5^v^	0.82	1.64	2.4486 (16)	170
N2—H2*A*···O3^v^	0.89 (3)	2.16 (3)	2.959 (2)	149 (2)
N2—H2*A*···O5^v^	0.89 (3)	2.36 (3)	3.007 (2)	130 (2)
N2—H2*B*···O3^vi^	0.89 (3)	1.99 (3)	2.870 (2)	173 (2)

Symmetry codes: (i) −*x*+2, −*y*+1, −*z*+2; (ii) −*x*+2, −*y*, −*z*+2; (iii) −*x*+1, −*y*, −*z*+1; (iv) −*x*+1, −*y*+1, −*z*+1; (v) *x*−1, *y*, *z*; (vi) −*x*+1, −*y*+1, −*z*+2.
